# Changing Microspatial Patterns of Sulfate-Reducing Microorganisms (SRM) during Cycling of Marine Stromatolite Mats

**DOI:** 10.3390/ijms15010850

**Published:** 2014-01-09

**Authors:** Alexandru I. Petrisor, Sandra Szyjka, Tomohiro Kawaguchi, Pieter T. Visscher, Robert Sean Norman, Alan W. Decho

**Affiliations:** 1 Department of Urban and Landscape Planning, School of Urban Planning, “Ion Mincu” University of Architecture and Urban Planning, str. Academiei nr. 18-20, sector 1, Bucharest 010014, Romania; E-Mail: alexandru_petrisor@yahoo.com; 2 National Institute for Research and Development in Constructions, Urbanism and Sustainable Spatial Development URBAN-INCERC, sos. Pantelimon, nr. 266, sector 2, Bucharest 021652, Romania; 3 Department of Environmental Health Sciences, Arnold School of Public Health, University of South Carolina, Columbia, SC 29208, USA; E-Mails: tkawaguchi@allenuniversity.edu (T.K.); rsnorman@mailbox.sc.edu (R.S.N.); 4 Center for Integrative GeoSciences, University of Connecticut, 345 Mansfield Rd., U-2045 Storrs, CT 06269, USA; E-Mail: pieter.visscher@uconn.edu

**Keywords:** biofilms, EPS, microbial mats, microspatial, sulfate-reducing microorganisms, *dsrA* probe, chemical signals, CaCO_3,_ AHLs, ^35^SO_4_^2−^ silver-foil

## Abstract

Microspatial arrangements of sulfate-reducing microorganisms (SRM) in surface microbial mats (~1.5 mm) forming open marine stromatolites were investigated. Previous research revealed three different mat types associated with these stromatolites, each with a unique petrographic signature. Here we focused on comparing “non-lithifying” (Type-1) and “lithifying” (Type-2) mats. Our results revealed three major trends: (1) Molecular typing using the *dsrA* probe revealed a shift in the SRM community composition between Type-1 and Type-2 mats. Fluorescence *in-situ* hybridization (FISH) coupled to confocal scanning-laser microscopy (CSLM)-based image analyses, and ^35^SO_4_^2−^-silver foil patterns showed that SRM were present in surfaces of both mat types, but in significantly (*p* < 0.05) higher abundances in Type-2 mats. Over 85% of SRM cells in the top 0.5 mm of Type-2 mats were contained in a dense 130 μm thick horizontal layer comprised of clusters of varying sizes; (2) Microspatial mapping revealed that locations of SRM and CaCO_3_ precipitation were significantly correlated (*p* < 0.05); (3) Extracts from Type-2 mats contained acylhomoserine-lactones (C4-, C6-, *oxo*-C6 C7-, C8-, C10-, C12-, C14-AHLs) involved in cell-cell communication. Similar AHLs were produced by SRM mat-isolates. These trends suggest that development of a microspatially-organized SRM community is closely-associated with the hallmark transition of stromatolite surface mats from a non-lithifying to a lithifying state.

## 1. Introduction

Microbial mats exhibit dense horizontal arrays of different functional groups of bacteria and archaea living in microspatial proximity. The surface mats of open-water marine stromatolites (Highborne Cay, Bahamas) contain cyanobacteria and other common microbial functional groups such as aerobic heterotrophs, fermenters, anaerobic heterotrophs, notably sulfate reducing microbes and chemolithotrophs like sulfur oxidizing microbes [1,2]. This community cycles through three different mat types and collectively constructs organized, repeating horizontal layers of CaCO_3_ (*i.e.*, micritic laminae and crusts), with different mineralogical features depending on community types [3,4].

Marine stromatolites represent dynamic biogeochemical systems having a long geological history. As the oldest known macrofossils on earth [5], extant marine stromatolites are still forming in isolated regions of shallow, open-water marine environments and are now known to result from microbially-mediated processes [4]. Stromatolites are ideal systems for studying microbial interactions and for examining mechanisms of organized biogeochemical precipitation of horizontal micritic crusts [4]. Interactions within and between key functional groups will be influenced, in part, by their microspatial proximities.

The surface microbial mats of Bahamian stromatolites are fueled by cyanobacterial autotrophy [6,7]. The surface communities of the mats repeatedly cycle through several distinct stages that have been termed Type-1, Type-2 and Type-3, and are categorized by characteristic changes in precipitation products, as outlined by Reid *et al*. [4]. Type-1 (binding and trapping) mats represent a non-lithifying, accretion/growth stage that possesses an abundant (and sticky) matrix of extracellular polymeric secretions (EPS) largely produced by cyanobacteria [8]. The EPS trap concentric CaCO_3_ sediment grains called ooids, and promote an upward growth of the mats. Small microprecipitates are intermittently dispersed within the EPS [9]. This accreting community typically persists for weeks-to-months then transforms into a community that exhibits a distinct bright-green layer of cyanobacteria near the mat surface. Concurrently the surface EPS becomes a “non-sticky” gel and begins to precipitate small patches of CaCO_3_. This morphs into the Type-2 (biofilm) community, which is visibly different from a Type-1 community in having a non-sticky mat surface and a thin, continuous (e.g., 20–50 μm) horizontal lithified layer of CaCO_3_ (*i.e.*, micritic crust). Type-2 mats are thought to possess a more-structured microbial biofilm community of sulfate-reducing microorganisms (SRM), aerobes, sulfur-oxidizing bacteria, as well as cyanobacteria, and archaea [2]. Studies have suggested that SRM may be major heterotrophic consumers in Type-2 mats, and closely linked to the precipitation of thin laminae [1,10]. The lithifying stage sometimes further progresses into a Type-3 (endolithic) mat, which is characterized by abundant populations of endolithic coccoid cyanobacteria *Solentia* sp. that microbore, and fuse ooids through dissolution and re-precipitation of CaCO_3_ into a thick contiguous micritized layer [4,10]. Intermittent invasions by eukaryotes can alter the development of these mat systems [11].

Over past decades a growing number of studies have shown that SRMs can exist and metabolize under oxic conditions [12–18]. Studies have shown that in marine stromatolites, the carbon products of photosynthesis are rapidly utilized by heterotrophic bacteria, including SRM [1,4,8,19]. During daylight, photosynthesis mat surface layers generate very high concentrations of molecular oxygen, mostly via cyanobacteria. Despite high O_2_ levels during this time, SRM metabolic activities continue [13,16], accounting for as much as ten percent of total SRM daily carbon requirements. During darkness HS- oxidation under denitrifying conditions may lead to CaCO_3_ precipitation [1,20]. Studies showed that concentrations of CaCO_3_ precipitates were significantly higher in Type-2 (than in Type-1) mats [21]. Using ^35^SO_4_ radioisotope approaches, Visscher and colleagues showed that sulfate reduction activities in Type-2 mats could be spatially aligned with precipitated lamina [10]. This has posited an important role of the SRM in the precipitation of laminae in Type-2 stromatolite mats. A similar role for SRM in precipitation of carbonate laminae has been described in lithifying hypersaline mats [22–24].

The development of a diverse, spatially-organized microbial community is often dependent upon interactions among its resident organisms and their physiochemical environment. Laboratory culture studies show that when bacteria are abundant and in spatial proximity they produce chemical signals, which are used to sense nearby cell densities and to coordinate gene expression among groups of cells in a process called quorum sensing [25]. More recently, a diverse array of chemical signals called acylhomoserine lactones (AHLs) were identified in the surface layers of stromatolite mats [26]. While quorum sensing is now a well-established process in laboratory cultures of bacteria, it is largely unexplored among the SRM [27] and its roles in natural communities are poorly understood [28,29].

Summarizing, SRM are likely to be an important regulatory component in the development and evolution of stromatolite mats [10], and in their precipitation of micritic crusts and laminae [1,22,23,30]. However, analyzing microspatial distributions of bacteria within intact microbial mats has been problematic. Here, we hypothesized that the SRM spatially organize in surface mats, communicate and coordinate activities using chemical signaling, and may be microspatially-associated with the precipitation of horizontal micritic crusts in Type-2 mats. Our study was designed to examine changes in the community, and *in situ* microspatial arrangements of SRM in non-lithifying (*i.e.*, Type-1) and lithifying (Type 2) stromatolite mats using fluorescence *in-situ* hybridization (FISH) probing coupled with confocal scanning laser microscopy (CSLM). Image-analyses, specifically using Geographical Information Systems (GIS) [31] and Digital image Analysis in Microbial Ecology (Daime; [32]) programs were employed to detect and compare changing microspatial arrangements of bacteria.

## 2. Results and Discussion

### 2.1. Overall Summary

Changes in the relative abundances and activities of specific functional groups of bacteria can be an important determinant for influencing elemental cycling and even the broader ecology of microbial mat systems [33]. In the open-water stromatolite mats of the Bahamas, the present study showed that the alternating stages of non-lithifying (Type-1) and lithifying (Type-2) surface mats possessed very different spatial distributions of bacteria, especially within the sulfate-reducing microorganism (SRM) clade. The classic Type-1 mats showed dispersions of cyanobacteria and heterotrophic bacteria, including the SRM, that were relatively random. As Type-1 mats transitioned into Type-2 mats the heterotrophic community, especially the SRM, became more abundant. Using GIS analyses, the area occupied by SRM cells in Type 2 mats was found to be double that of their Type-1 counterparts. Cells also became more microspatially-organized in Type-2 mats. This was accompanied by an increased frequency of cell clustering. Further, the relative sizes of clusters increased in Type-2 mats, eventually forming an almost contiguous thin (130 μm) horizontal layer of SRM at the uppermost mat surface. This suggested an increasing community organization. Development of this dense heterotrophic layer was concurrently associated with precipitation of CaCO_3_, the production of cell-cell chemical signals, and a dramatic shift in the phenotypic properties of the mats.

### 2.2. Properties of Type-1 and Type-2 Mats

**Figure 1.**
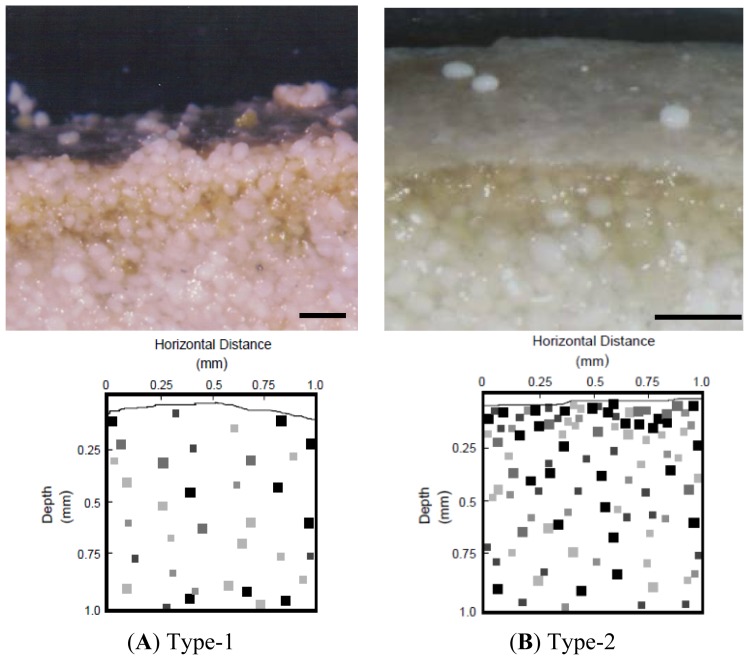
Light micrographs of cross-sections showing surfaces of Type-1 and Type-2 stromatolite mats. Light micrographs of a Type-1 mat (**A**) show an irregular “sticky” EPS-laden surface that accretes ooid grains, while the Type-2 mat (**B**) is characterized by a “non-sticky, white precipitate” crust on the surface. Three ooids have been artificially placed on the Type-2 surface crust to further illustrate the precipitate. Scale bars = 500 μm. Lower panels show 2D images 1 × 1 mm in size of the surface of both mats (light grey line indicates the mat surface). Images were generated from ^35^SO_4_^2−^ silver (Ag) foil experiments. Mat cross-sections were incubated on silver foil impregnated with the sulfate radioisotope. SRM reduce the ^35^SO_4_^2−^ to ^35^S^2−^, which precipitates as Ag^35^S is was visualized with radiography. Black pixels indicated areas of intense sulfate reducing activity.

Light microscopy examinations of mat surfaces showed that Type-1 mats of stromatolites were characterized by an irregular and adherent surface (*i.e.*, Type-1 mat; Figure 1A), which collects sediment grains (*i.e.*, carbonate ooids) within a matrix of extracellular polymers (EPS). The EPS matrix is known to enhance light penetration into the mat [34]; a process that is associated with the physical stabilization of the mat since EPS often increases the cohesive properties of sediments [35]. Oxygen profiles show a diffuse zone of photosynthesis and ^35^SO_4_^2−^-labeled silver (Ag) foils indicated few SRM were present in the upper mm of the mat (Figure 1A, lower panel). This was followed by the appearance of a thin (30–50 μm thick) crust of CaCO_3_ precipitate (*i.e.*, Type-2 mat; Figure 1B). The macroscopic appearance of the two types of mat surfaces was easily distinguishable under low magnification (*i.e.*, 70–150×) using a dissecting microscope.

### 2.3. *dsrA* Oligoprobing

Our study utilized the *dsrA* oligoprobe to conservatively target SRM, including the sulfate-reducing bacteria. Sulfate reduction is known to occur in a wide range of bacteria, and some Archaea [36,37]. Through examinations of intact mat sections, and the coupling of fluorescence in situ hybridization (FISH) with confocal scanning laser microscopy (CSLM), and geographical information systems (GIS) analyses, it was possible to examine the *in situ* organization of SRM cells over microspatial scales and how the organization of this microbial functional group changed in different mat types within the stromatolite system. We showed that SRM were present in the upper-most surface layers of both Type-1 and Type-2 mats. However, within Type-1 mats, SRM cell abundances were comparatively lower, and SRM cells were relatively randomly dispersed within the EPS matrix. This was confirmed by the ^35^SO_4_^2−^-Ag foil observations (Figure 1B, lower panel). In contrast, distributions of cells within Type-2 mats showed that SRM became increasingly more abundant and more-clustered in their distribution, especially within the uppermost mat surface. The *dsrA* probe and ^35^SO_4_^2−^-Ag patterns are both in agreement for Type-2 mats as well.

The use of fluorescently-labeled rDNA oligo-probes for determinations of specific microbial cells in complex media presents several inherent obstacles [38,39]. The first relates to non-specific binding of probes in the complex media. Second, the signal intensity of a given cell is directly linked to ribosomal content and hence physiological activities of cells at the time of fixation. However, oligoprobes can be very useful for evaluation of changing spatial patterns of microorganisms [39,40]. To further examine the specificity of our *dsrA* oligoprobe, sections of Type-1 and Type-2 mats were imaged at higher magnifications (e.g., 600× to 1000×). Co-localized fluorescence of the oligoprobes (indicative of SRM cells) and also DAPI (4′,6-diamidino-2-phenylindole, dihydrochloride) or PI (propidium iodide) were used to determine cell-specific binding of oligoprobes and to remove non-specific fluorescence signatures. Hence, cell areas containing both fluorescence signatures were counted as SRM cells. This allowed us to reduce the effects of non-specific binding of oligoprobes, and to digitally remove most of the non-specific binding effects in estimations of cell abundances.

### 2.4. Relative Abundances of SRM

Significantly (*p* < 0.05; Student’s *t*-test) higher abundances of SRM cells were observed in the surfaces of Type-2 mats when compared with Type-1 mats. Using geographical information systems (GIS) analyses, abundances of cells were determined as a function of “fluorescence area” occupied by SRM cells relative to other fractions of the microbial community. Statistical analyses (Student’s *t*-test) compared the portion of the total microbial community that was SRMs located within the top 130 μm of the two mat types. Appropriate transformations were made, where necessary, to normalize data for parametric tests. Relative abundances of SRMs in surfaces of Type-1 and Type-2 mats were expressed as a mean (±SE) percent (%) of total cell areas attributable to SRM within the uppermost 130 μm of the mats. Results of a student *t-*test showed the surfaces of Type-2 mats (88.0% ± 14.2%; *n* = 31 images analyzed) contained a significantly (*p* < 0.0001) higher abundance of cells (based on cell area) than Type-1 mats (39.7% ± 27.5%; *n* = 21). The results indicated that as the Type-1 community transitions into a Type-2 community, a significantly larger proportion of the total bacteria community (in Type-2 mats) were SRM.

#### 2.4.1. SRM as Portion of Total Microbial Cells

Using direct counts of DAPI-stained cells we further confirmed that higher abundances of all microbial cells (*i.e.*, SRM, other bacteria, archaea) occurred in surfaces of Type-2 mats, when compared with Type-1 mats. The SRM comprised greater than half of the total microbial cells extractable from surface Type-2 mats. When cells were extracted from Type-2 mats and direct counts were estimated using either DAPI-staining or propidium-iodide-staining and compared to SRM cell counts using *dsrA*-staining, the SRMs represented 55.9% ± 20.0% and 56.1% ± 16.2% (mean ± SE), respectively, of the total bacteria cells detected. In contrast, SRM cells in Type-1 mats (as estimated using *dsrA*) comprised only 20.7% ± 9.3% of the total microbial cells. These observations were confirmed by the ^35^SO_4_^2−^-Ag foil observations that documented a 2D distribution of sulfate reducing activity (Figure 1; [10]).

**Figure 2.**
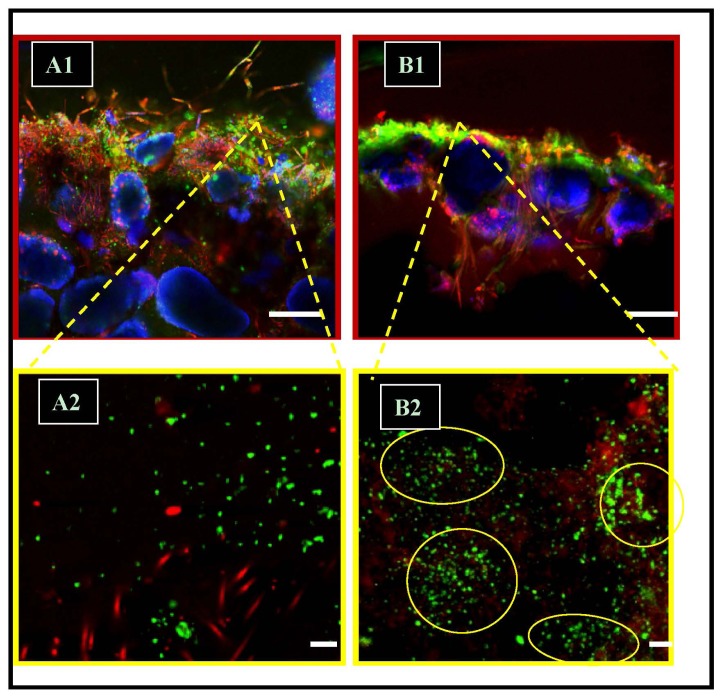
Confocal scanning laser micrographs (CSLM) illustrating relative changes microspatial distributions of SRM cells near the surface of (**A1**, **A2**) Type-1 (*i.e.*, relatively-scattered) and (**B1**, **B2**) Type-2 (*i.e.*, highly-clustered) mats. Images are cross-sections of surface mats showing SRM cells (green fluorescence; *dsrA* FISH probe), heterotrophic bacteria (red fluorescence stained with propidium-iodide (PI)) and cyanobacteria (red autofluorescence), and ooid sediment grains (artificial blue-color). Yellow circles illustrate typical clustering of SRM cells. Scale bars in **A1** and **B1** = 100 μm; in **A2** and **B2** = 10 μm.

Image analyses revealed interesting spatial patterns of bacteria. Images were collected from cross-sections of surface mats and focused analyses from the immediate mat surface to approximately 0.75 mm depth. Additionally, we analyzed spatial variability of the surface over a full horizontal distance of 850 μm. This allowed us to examine two-dimensional spatial patterns (e.g., horizontal layering, clustering, and dispersion) over relatively large regions of the uppermost surface of Type-1 and Type-2 mats (Figure 2A1, B1). Higher magnifications (1000×) were then used to examine smaller scale (e.g., 1 to 50 μm) patterns and clustering of cells (Figure 2A2, B2).

### 2.5. Precipitation Patterns: Microspatial Associations of SRMs and Precipitates

A highly-significant (*p* < 0.05; Student’s *t*-test) statistical difference was detected in the areas occupied by precipitates. Results showed that precipitates were less abundant, in terms of area, in Type-1 mats when compared with Type-2 mats.

**Figure 3.**
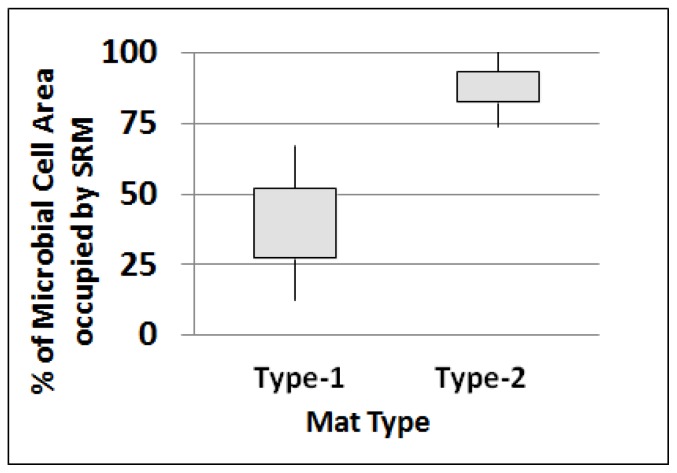
Box-plot showing the percent of area occupied by all microbial cells, which were SRM. Results show that in Type-2 mats, over 80% of microbial cells (based on area occupied) were SRM. Note: Type-1 mats (*n* = 21) and Type-2 mats (*n* = 31); tails represent 95% confidence intervals (CI).

Based on the assumption that precipitation of CaCO_3_ was related to SRM activities, we examined the microspatial locations of SRM cells and CaCO_3_ precipitates within images from both Type-1 and Type-2 mats. A significant (*p* < 0.05) correlation (*r* = 0.757) was found linking SRM and CaCO_3_ precipitates within the same image (*n* = 34). In both Type-1 and Type-2 mats, there was a close microspatial association of SRM cells and CaCO_3_ precipitates with SRMs constituting over 80% of microbial cells that were located within a 4.4 μm distance of precipitates (Figure 3). Most of these cells occurred within a 1.1 μm distance (Table 1). This is noteworthy because although precipitates occur to a limited extent in Type-1 mats, SRM were still closely-associated with the precipitates that were present. This suggested a close relationship of SRMs and the precipitation process in both mat types.

It is important to note that in observing both Type-1 and Type-2 natural mats, variability existed over small spatial scales in the patterns of cells and precipitation products. This is likely a result of the localized interactions between bacteria and their environment. While this variability may be adaptive, in an ecological sense, it resulted in having to examine a large number of images to acquire sufficient statistical power for examination of potential differences (if present). Examination of the vertical distribution of SRMs situated within the top 500 μm indicated that the majority (over 85%) of SRM cells were located in the top 130 μm of the surface of Type-2 mats. These results suggest that SRM distributions may be used as an instrument of discrimination for categorization between Type-1 and Type-2 mats, with higher surface abundances of SRM occurring in Type-2 mats.

### 2.6. Phylogenetic Analysis of the *dsrA* Sequences

**Figure 4.**
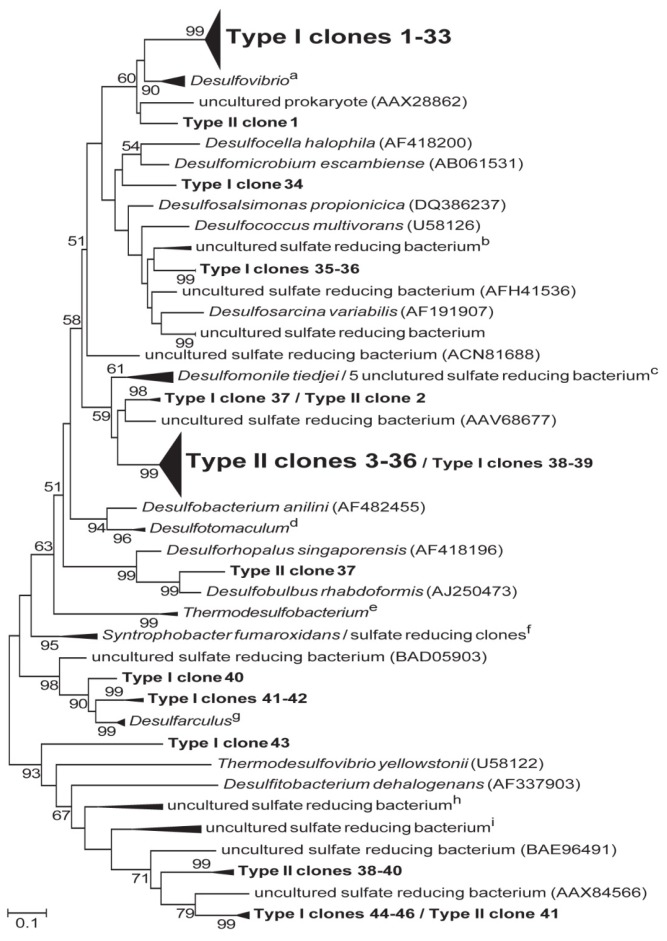
Phylogenetic tree based on translated amino acid sequences of PCR-amplified dissimilatory sulfite reductase *dsrA* genes retrieved from type I and type II stromatolites. Tree shows distributions of clones related to known sulfur-reducing bacteria and closely related sequences obtained from the GenBank database. GenBank accession numbers are shown in parentheses for non-collapsed branches and are as follows for collapsed branches: ^a^ AFA43406, EU127914, BAB55577, AFA43404, BAB55579, AB061543; ^b^ ACI31420, ABK90679; ^c^ ABK90745, AF334595, ABK90741, ABK90691, AAO61116, ABK90759; ^d^ AF271769, AF273029; ^e^ AF271771, AF334598; ^f^ AF418193, CAY20641, CAY20696; ^g^ YP003806924, AAK83215, AF334600; ^h^ AEX31202, CAJ84858, CAQ77308; ^i^ ACJ11472, CAJ84838, ACJ11485, ABK90809. The tree was constructed using the maximum likelihood method in MEGA 5 with values at nodes representing bootstrap confidence values with 1000 resamplings. Bootstrap values are shown for branches with more than 50% bootstrap support. Scale bar represents 0.1 substitutions per site.

Phylogenetic relationships of *dsrA* gene sequences retrieved from Type-1 and Type-1-2 stromatolite mats revealed an overall low diversity (Figure 4). Type-1 *dsrA* clone sequences formed 9 different phylogenetic groups with nearly 72% of clone sequences located in a single clade most similar to *dsrA* genes of the Gram-negative delta-proteobacteria *Desulfovibrio*. Type-2 *dsrA* clones formed 6 different phylogenetic groups with nearly 83% of all clone sequences located in a single clade most similar to the delta-proteobacteria *Desulfomonile tiedjei* and other uncultured SRM capable of autotrophic growth. Most of the few remaining *dsrA* clone sequences formed monophyletic lineages that were distinct for either Type-1 or Type-2 stromatolite mats and included sequences similar to the deeply branching *Thermodesulfovibrio yellowstonii* and other uncultured sulfate-reducing bacteria. Preliminary 16S rDNA investigations of SRM diversity in a hypersaline lake with lithifying and non-lithifying mats [22], showed a dominance of delta-proteobacteria (91% and 64% of total diversity in lithifying and non-lithifying mats, respectively [2]. In this study, a wider diversity of delta-proteobacteria was observed in the lithifying mats when compared to non-lithifying mats and SRM activity was associated with the upper layer of the mats that were forming a CaCO_3_ crust. This suggests that patterns observed in this study could apply to other lithifying systems as well.

### 2.7. Microspatial Clustering Analyses

Clustering, defined here as the aggregation of cells in spatial proximity, is likely an important parameter for assessing the microbial communities of stromatolites. When microbial cells are clustering together in proximity it increases their ability to interact in both positive and negative manners. Such clusters may provide a suitable proxy indicative of chemical communications, such as quorum sensing (QS) [25] and/or efficiency sensing [41]; processes that bacteria and other microorganisms likely utilize under natural conditions, especially within biofilms (e.g., microbial mats). SRM are physiologically challenged by the exposure to high O_2_ levels at the surface of the mats where their activity peaks (see [2] for review). It is thought that this high activity is supported by abundant organic carbon, especially low-molecular weight compounds [8,19]. Recently QS signals have been extracted from marine stromatolite mats [26]. QS signals could be correlated with SRM and were postulated to play an important role in enabling these anaerobes to cope with O_2_ concentrations that are deleterious to their physiology [42]. QS contributes to the coordination of gene expression and metabolic activities by neighboring cells, and may play important roles in the development of microbial consortia under natural conditions [42]. In other systems, QS signaling has been shown to be detectable by cells at distances extending up to 73 μm [43]. A second benefit of chemical communication resides in efficiency sensing, often considered an extended form of quorum sensing. Efficiency sensing, however, provides cells with the ability to assess the diffusional properties of their proximal extracellular environment [41]. Finally, clustering invokes a new (and smaller) spatial scale perspective for understanding the formation of sharp geochemical gradients and the efficiency of elemental cycling that are characteristic of mats.

We were able to show that SRM showed little- or no-clustering in Type-1 mats but that very well-developed clustering occurred in Type-2 mats. The rapid upward growth (accreting) nature of Type-1 mats may not allow for such spatial organization to develop. The microspatial organization of cells into clusters (*i.e.*, groups of cells in proximity) was discernible at several spatial scales. Imaging using CSLM was coupled to the general labeling of cells using DAPI and PI, and more specific labeling using FISH targeting the SRM group. Using this approach, two different spatial scales of clustering became detectable. At relatively low magnifications (e.g., 200×) the distinctly higher abundances of SRMs were easily visualized near the surface of Type-2 mats (Figure 2). The non-lithifying Type-1 mats exhibited lower abundances and a relatively “random” distribution of SRM, and other bacteria, when compared with the non-random organization of bacteria in Type-2 mats. Overall differences determined by ANOVA were significant (*F* = 33.55, *p* ≤ 0.05). All aposteriori specific tests (Bonferroni, and Scheffé) placed Type-1 different from the Type-2 mats, the latter of which exhibited significantly greater abundances of SRMs. At higher magnifications it became apparent that the Type-2 mat community exhibited an increase in clustering and microspatial organization, especially with regard to the SRM functional group (Figure 2). The frequency of SRM cell clusters increased, when compared with Type-1. Finally, the mean size (and variance) of clusters also increased as mats develop from a Type-1 to a Type-2 state, implying that some clusters became quite large. This occurred in the uppermost 50 μm of the surface biofilm.

These patterns were supported by image analyses using GIS [44] and Daime [32,45] programs and resulted in statistically (*p* < 0.001) higher abundances of SRM in the surfaces of Type-2 mats (when compared with Type-1). Two different, but complementary, methodological approaches (*i.e.*, Daime and GIS) were used in this study to detect microspatial clustering of cells.

#### 2.7.1. The Daime Approach

**Figure 5.**
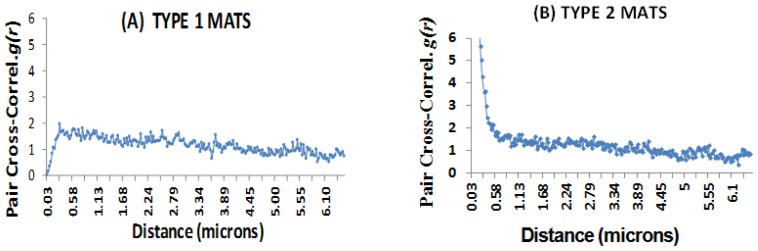
Microspatial clustering arrangements of SRM cells located in the surfaces of stromatolite mats using *Daime* analyses. The graphs exhibit the pair cross-correlation function *g*(*r*) for SRM cells. (**A**) In Type-1 mats, the relatively horizontal line where *g*(*r*) approximates 1 indicates relatively random SRM distributions over cell-cell distances ranging from 0.1 to 6.44 μm; (**B**) In Type-2 mats, values of *g*(*r*) above 1 indicate a high degree of clustering of SRM cells, especially over short (e.g., 0.03 to 0.36 μm) cell-to-cell distances. This indicates that cells in Type-2 mats are clustered closely together.

The first approach, the Daime program [32], allowed us to examine all cell-cell distances within an image and graph the distances. Analyses of SRM spatial arrangements showed that in Type-1 mats (Figure 5A), the pair cross-correlation index *g*(*r*) was close to 1 for cell-to-cell distances ranging from 0.1 to 6.44 μm, which is indicative of a relatively random distribution. A flat line (*r* = 1) was indicative of a relatively random distribution, where all cell-cell distances were equally probable. In Type-2 mats (Figure 5B), by contrast, the pair cross-correlation index was above 3 at a distance 0.36 μm, and rose to 52 at cell-cell distances of 0.03 μm. These data indicated that the SRM had a high degree of clustering, especially where cell-cell distances were very short. It can be inferred from these data that clusters were abundant in Type-2 mats and that the cells within SRM clusters were in very close proximity (*i.e.*, from 0.03 to 0.36 μm). Overall, when comparing cell distributions in Type-1 and Type-2 surface mats, there was increased clustering observed in Type-2 mats.

#### 2.7.2. The GIS Approach

A second approach utilized GIS examined clustering of SRM cells within the surfaces of Type-1, and Type-2 mats. For each image a buffer area was created that extended from the surface of the mat to approximately 130 μm depth. Detection of SRM cells within the buffer area was based on color (as described above) using image classification of FISH-probed cells. A concentric region having a 10 μm diameter was generated around each cell. A cluster represented a group of cells having overlapping concentric regions. Subsequent statistical selection of clusters was subjectively based on cluster areas representing greater than five cells having overlapping concentric regions. The size (*i.e.*, area) of each detected cell cluster was measured. While the two methods utilize different approaches to detect clustering, both revealed a similar inference-increased clustering present in Type-2 mats.

**Figure 6.**
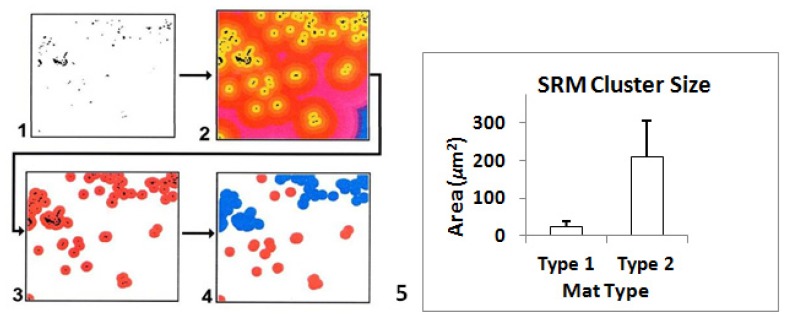
Scheme illustrating detection of SRM clusters using GIS. (**1**) CSLM micrograph showing SRM cells labeled with *dsrA* probe with background digitally-removed, and identification of individual SRM cells (*i.e.*, black dots); (**2**) generation of artificial concentric regions with same width (10 μm) around each cell or group of cells; (**3**) identification of overlapping concentric regions; (**4**) statistical selection of clusters based on area (e.g., overlapping areas of > five cells); (**5**) Graph showing cluster sizes of SRM cells in Type-1 and Type-2 mats. Means and 95% confidence intervals are expressed as areas for SRM clusters. Note the significantly larger sizes and variability in cluster-sizes detected in Type-2 mats.

Finally, the size distribution of SRM clusters (including individual cells) was statistically analyzed using samples of 20 images that were randomly selected from microspatial regions within images from each mat type (Type-1, Type-2, and incipient Type-2) labeled with the *dsrA* oligoprobe. Type-2 exhibits the largest clusters (Figure 6). The mean cluster size was comparatively small in Type-1 mats and large in Type-2 mats. Variability followed the same pattern, increasing from Type-1 to Type-2.

#### 2.7.3. Image Analyses

Proper image interpretation was needed to examine microscopic spatial patterns of cells within the mats. We employed GIS as a tool to decipher and interpret CSLM images collected after FISH probing, due to its power for examining spatial relationships between specific image features [46]. In order to conduct GIS interpolation of spatial relationships between different image features (e.g., groups of bacteria), it was necessary to “ground-truth” image features. This allowed for more accurate and precise quantification, and statistical comparisons of observed image features. In GIS, this is typically accomplished through “on-the-ground” sampling of the actual environment being imaged. However, in order to “ground-truth” the microscopic features of our samples (and their images) we employed separate “calibration” studies (*i.e.*, using fluorescent microspheres) designed to “ground-truth” our microscopy-based image data.

Quantitative microspatial analyses of *in-situ* microbial cells present certain logistical constraints that are not present in the analysis of dispersed cells. In the stromatolite mats, bacterial cells often occurred in aggregated groups or “clusters”. Clustering of cells needed evaluation at several spatial scales in order to detect patterns of heterogeneity. Specifically, we wanted to determine if the relatively contiguous horizontal layer of dense SRM that was visible at larger spatial scales was composed of groups of smaller clusters. We employed the analysis of cell area (fluorescence) to examine *in-situ* microbial spatial patterns within stromatolites. Experimental additions of bacteria-sized (1.0 μm) fluorescent microspheres to mats (and no-mat controls) were used to assess the ability of GIS to “count cells” using cell area (based on pixels). The GIS approach (*i.e.*, cell area-derived counts) was compared with the direct counts method, and product moment correlation coefficients (*r*) were computed for the associations. Under these circumstances the GIS approach proved highly useful.

In the absence of mat, the correlation coefficient (*r*) between areas and the known concentration was 0.8054, and the correlation coefficient between direct counts and the known concentration was 0.8136. Areas and counts were also highly correlated (*r* = 0.9269). Additions of microspheres to natural Type-1 mats yielded a high correlation (*r* = 0.767) between area counts and direct counts. It is realized that extension of microsphere-based estimates to natural systems must be viewed conservatively since all microbial cells are neither spherical nor exactly 1 μm in diameter (*i.e.*, as the microspheres). Second, extraction efficiencies of microbial cells (e.g., for direct counts) from any natural matrix are uncertain, at best. Hence, the empirical estimates generated here are considered to be conservative ones. This further supports previous assertions that only relative abundances, but not absolute (*i.e.*, accurate) abundances, of cells should be estimated from complex matrices [39] such as microbial mats.

Results of microbial cell estimations derived from both direct counts and area computations, by inherent design, were subject to certain limitations. The first limitation is inherent to the process of image acquisition: many images contain only portions of items (e.g., cells or beads). In terms of counting, fragments or “small” items were summed up approximately to obtain an integer. Therefore, the solution used was the “counting rule”. The problem disappears when total areas are computed. A second limitation involves image overlap [47]. This problem affects the computation of areas in the absence of a mathematical model that would account for overlapping objects. The human eye, for example, can readily distinguish between overlapping beads, and as a result traditional counting was less affected. While area computations were slightly influenced by this, the solution was approached in the same fashion as above (*i.e.*, through direct count comparisons) and the results were comparable. A third limitation relates to the three-dimensional nature of samples. Items situated slightly below the plane of focus sometimes produce residual fluorescence and appear as smaller items of the same kind or fragments. While those items might have been counted during direct counts, it was difficult to generate an objective means (*i.e.*, a systematic counting rule) to account for such items. A simple solution, however, was obtained when areas were computed during image analysis. The solution resided in the image classification process. Items situated below the plane of focus fluoresced at a lower intensity. Based on the threshold value some of them were classified as background and eliminated from computations, while others were registered as items of interest. As a result, area

Computation incorporated a systematic approach to overcome this difficulty. Finally, the GIS-based approach was proposed as an alternative to the direct-counts method or other methods, and not as a replacement. Statistical analyses indicated that there were no significant differences between the direct counts and GIS methods when used to estimate the concentrations of microspheres, and area computations using GIS represented a successful alternative for estimating relative abundances of microbial cells in this mat system, especially at high cell abundances.

### 2.8. Ground-Truthing GIS at Microbial Spatial Scales

#### 2.8.1. Fluorescent Microsphere Additions to Type 1 Mats

Results from analyses between areas of microspheres computed (via GIS) for each image individually and the total number of microspheres counted within the same image using, showed a highly-significant (*p* < 0.0001) product moment correlation coefficient (*r* = 0.767).

### 2.9. AHL Chemical Signals within Type-2 Mats

The high abundances of SRM cells underscore the potential impact of this clade on the mat system. The process of cell–cell chemical communication, called quorum sensing, facilitates coordination of group activities, and is now realized to play important roles in natural microbial communities [25–29]. Given the importance of sulfate reduction across many environments, it is therefore surprising that few reports exist for quorum sensing within the sulfate reducing clade, either within the delta proteobacteria [27] or the archaea. This earlier study [27] noted production of several AHLs by a stromatolite mat isolate of *Desulfovibrio* sp. (strain H2.3jlac), one of the same strains examined in this study. We examined two additional strains of SRB isolated from a Type-2 stromatolite mat: *Desulfovibrio* strain H2.3jman (isolated on mannose as the electron donor) and *Desulfovibrio* strain

**Figure 7.**
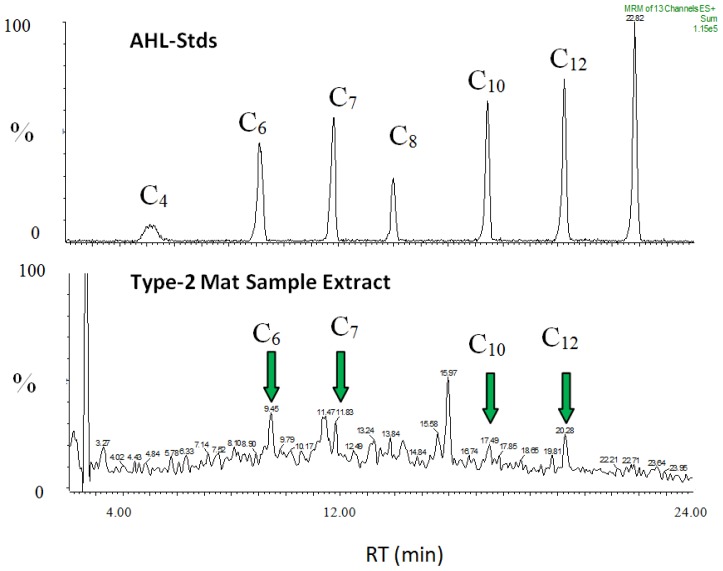
Spectra showing AHLs extracted from Type 2 mats, and AHL standards. Samples are separated using LC/MS. Peaks are shown as a relative percent (*y*-axis), while *x*-axis shows retention time (RT), expressed in minutes.

H12.1lac (isolated on lactate as electron donor). Both strains also produced a wide range of AHLs (e.g., C6, C7, C8, C10) under standard culture conditions (Table 2, Figure 7). These are the same molecular congeners of AHL signals that were extracted from our natural mats, where high abundances of SRM were found.

The observed high abundances and clustering of microbial cells, coupled to the three-dimensional EPS matrix present within mats provide an ideal landscape to foster chemical communication among microbial cells, especially within Type-2 mats. The abundant SRM cell clusters, which were observed in the uppermost surfaces of the Type-2 mats using CSLM, present an ideal location for quorum sensing to occur in the mat. Under the natural conditions within microbial mats and the diffusional constraints related to EPS, quorum sensing among cells is likely to efficiently occur over relatively small spatial scales (e.g., 10’s of μm). Interestingly the sizes of SRM clusters, which we measured in Type-2 mats, also occurred within this size range. It must be emphasized, however, that a single mat sample (sample core area = 5.07 cm^2^) used for signal analyses contains a multitude of microbial clusters. Thus the microspatial variability of AHL signals could not be addressed here.

#### 2.9.1. SRM in Oxic Environments and CaCO_3_ Precipitation (Relevance)

Previous microelectrode studies have shown that the surfaces of both Type-1 and Type-2 mats were highly-oxygenated during daylight [10,48], with O_2_ concentrations in stromatolites reaching over 600 μM during peak photosynthesis [26]. While O_2_ has been classically considered to be stressful to most SRM [18], abundant populations of different SRM are now known to occur in oxygenated environments that display maximum metabolic rates under these conditions [12,14,49,50]. High abundances of SRM and sulfide-oxidizing microbes (SOM) were reported for the Highborne Cay stromatolites, and associated with this were high rates of sulfate reduction and sulfide oxidation [1]. Interestingly, this study found higher abundances and metabolic rates associated with lithifying layers (*i.e.*, Type-2 mats) than with non-lithifying layers (*i.e.*, Type-1 mats). A similar scenario was described for non-lithifying and lithifying mats in a hypersaline pond in the Bahamas, where higher cell densities and metabolic rates of sulfur-cycling organisms were associated with the mats that precipitated CaCO_3_ [2,22]. While the SRM in the current study occurred in the uppermost surface (*i.e.*, top 130 μm) of Type-1 mats, they were significantly denser and more clustered in Type-2 mats. These data suggest that significant sulfur cycling may be occurring within the upper mm of stromatolite mats. A fundamental question guiding a theoretical understanding of stromatolite formation is: Why do SRMs tend to aggregate at the surface of Type-2 mats? Several possibilities exist to explain the occurrence of SRM at the mat surface: (1) The surface of a Type-2 mat is underlain by a dense layer of cyanobacteria, and hence, is highly-oxic during approximately half the day of each diel cycle. The SRM may receive photosynthetic excretion products from cyanobacteria on a diel basis [8]. It is postulated here that they precipitate a CaCO_3_ cap to reduce DOC loss to the overlying water (which is oligotrophic), or to enhance efficient recycling of nutrients (e.g., N, P, Fe, *etc*.) within the mat. (2) A second possibility is that the SRM are physiologically adapted to metabolize under oxic conditions part of the time. Studies by Cyprionka [18] and others [2,51] have shown that some SRM may be physiologically adapted to cope with high O_2_ levels. In this case, CaCO_3_ precipitation could be advantageous as it produces a cement layer that increases the structural integrity of the stromatolite.

#### 2.9.2. A Broader Role of Cell Clustering in Microbial Landscapes

Biofilms have been described as microbial landscapes owing to their physical, metabolic and functional diversity [52]. Our results emphasize that the microspatial patterns of cells within the surface biofilms of marine stromatolites may exist at several different spatial scales: (1) Micro-scale (μm) clustering, which may occur as a few (e.g., 2–5) to hundreds of cells within a single cluster. Such clustering may facilitate regulation of group activities, such as quorum sensing; (2) Aggregation of clusters: Clusters themselves may aggregate (*i.e.*, merge with adjacent cell clusters) to form a horizontal layer, within a vertical geochemical gradient region of the mat; (3) Larger mm-scale layering: The visible (to the eye) horizontal zonations, which are indicative of major functional clades within microbial mats, contribute to the exchange of autotrophically-generated DOC to heterotrophs and efficient recycling to reduce loss of DOC to overlying water. QS may be used for coordination of inter- and intra-species metabolic activities, as suggested by Decho and colleagues [42]. In the specific case of SRM, which rely on cyanobacteria for DOC but are negatively affected by the O_2_ these phototrophs produce, it is of utmost importance to coordinate physiologies (including metabolisms) with other microorganisms that remove O_2_ during their metabolism. This role could be fulfilled by aerobic heterotrophs and SOM, the latter benefitting from optimal SR activity to provide the substrate for sulfide oxidation. Especially noteworthy is that sulfide removal by SOM also benefits cyanobacteria, for which high concentrations of sulfide are toxic. Coordination of metabolisms may be facilitated by QS in this case. Inter-specific QS may ultimately be a key process in shaping the biofilm architecture. This is currently under investigation.

## 3. Experimental Section

### 3.1. Sampling of Intact Mats

All stromatolite sampling was conducted at a subtidal marine environment site at Highborne Cay, Exumas, Bahamas (76º51′W; 24º42′N). The site has been under long-term investigation through the Research Initiative on Bahamian Stromatolites (RIBS) project [4]. Freshly-collected intact stromatolites were dissected into working samples (approx. 2 × 2 cm), then immediately fixed (overnight, 4 ºC) in a 4% paraformaldehyde (35 ppt seawater; 0.2 μm-filtered) solution. Portions of mat samples were initially trimmed into thick (approx. 2–4 mm) cross-sections using a rock saw, gently washed, and placed on glass microscope slides. Samples were then prepared for FISH. Surface mats were tentatively identified, based on light-microscopy examination of precipitation products, as either “Type-1” (*i.e.*, no visible surface precipitation), or “Type-2” (*i.e.*, crusty surface precipitation of CaCO_3_ present) mats (Figure 1). Samples within each mat type were pooled. The samples were used to examine *in situ* distributions of cells within mats. Samples that were in-transition between full Type-1 or Type-2 were not considered further.

### 3.2. Fluorescence *in-Situ* Hybridization (FISH)

The oligodeoxynucleotide probe *dsrAB* was custom-synthesized by GeneDetect (Aukland, New Zealand) using sequences from the 16S rDNA oligonucleotide ProbeBase [53,54]. The probe *dsrAB* (GD1001-CS with GreenStar *™ FITC fluorescent labeling, Molecular Probes, Eugene, OR, USA) was used to target the dissimilatory sulfite reductase genes (*dsrAB*) of all recognized lineages of sulfate-reducing bacteria and archaea [36,38,55]. The probe was composed of a cocktail of the *DSR1F* (sequence: ACS CAC TGG AAG CACG) and the *DSR4R* (sequence: GTG TAG CAG TTA CCG CA) primers [38,56,57]. Concentrations of *dsrAB* were 5 ng per μL, and appropriate nonsense controls were used. Hybridization mixtures were removed and slides were washed for 15 min, in buffer containing 20 mM Tris-HCl (pH 7.4), 0.225 M NaCl, and 0.01% SDS. Fluorescence signals were amplified using the Alexa Fluor 488 Signal-Amplification Kit (Molecular Probes, Eugene, OR, USA) for Oregon Green Dye-Conjugated Probes (Molecular Probes, Eugene, OR, USA). DAPI (4′6′-diamidino-2- phenylindole) and PI (Molecular Probes, Eugene, OR, USA) were also used for general bacteria (DNA) staining [58,59]. FISH-probing was conducted according general methods modified from [60–62]. After fixation, intact mat samples were gently washed in phosphate-buffered saline (PBS) and stored in ethanol:PBS (1:1) at −20 ºC. Samples, sliced into 2–4 mm sections on glass slides, were immersed in an ethanol series (50%, 80%, and 96%) for 3 min each. *In situ* hybridizations were performed at 50 ºC overnight in a hybridization buffer containing 0.9 M NaCl, 20% formamide, 20 mM Tris-HCl (pH 7.4), and 0.01% sodium dodecyl sulfate (SDS).

### 3.3. Extraction of Bacterial Cells from Mat Slurries

Cells were extracted from the mat matrix using additional samples. This approach was conducted to determine the portion of total (extractable) cells (*i.e.*, DAPI-stained or PI-stained cells) that hybridized using the FISH probes (*i.e.*, SRM cells). Samples from the uppermost surface mats were fixed in 4% buffered paraformaldehyde overnight at 4 ºC. The mat was gently homogenized into sediment slurries, then suspended in pre-filtered (0.2 μm) seawater. Cells were initially separated from sediment particulates using gentle centrifugation (1500× *g*; 2 min). Following, the cells and other organics (e.g., EPS) contained in the supernatant, were removed and subjected to repeated centrifugations (16,000× *g*; 10 min each) to pellet cells, and shear off EPS and other organics. The fixed, extracted cells were washed three times with 1× PBS (phosphate buffered saline), and stored in PBS/ethanol (1:1) at −20 ºC until further processing. Cells, contained in wells on slides, were incubated at 46 ºC for 90 min. in a hybridization buffer containing 0.9 M NaCl, 20% formamide, 20 mM Tris-HCl (pH 7.4), and 0.01% sodium dodecyl sulfate (SDS). The *dsrAB* probe concentration for slurry cell incubations was 1.0 ng per μL. Hybridization mixtures were removed and the slides were washed for 15 min, in buffer containing 20 mM Tris-HCl (pH 7.4), 225 mM NaCl and 0.01% SDS. Washing buffer was removed and washed with distilled water, and slides were air dried. Then, 50 μL of DAPI (or PI) was added on slides and incubated for 3 min. After washing with 80% ethanol, to remove unspecific staining, cells were rinsed in distilled H_2_O and air-dried. The slides were mounted with Citifluor (Citifluor Ltd., Canterbury, UK) and the oligo-probed cells were quantitatively imaged.

### 3.4. Confocal Scanning Laser Microscopy (CSLM)

Images were obtained using a CSLM system (Leica TCS SP5, Leica Microsystems, Germany) equipped with a Kr-Ar laser. For CSLM imaging, three internal detectors were used, each with a 6-position emission filter wheel and a variable confocal aperture. Sample slides were viewed using 20×, 40×, 60×, or 100× objectives. The 60× and 100× objectives were used with immersion oil (Stephens Scientific Co., # M4004; Riverdale, NJ, USA; refractive index 1.515) to image individual cells. Final output was represented by colored composite images exported in a tagged image file format (TIFF). Direct counting of DAPI-stained cells and the oligoprobe-hybridized cells were performed on images of 30 independent fields using the automated image analysis software, Cell-C program [63]. In this manner, the relative proportions of SRM: total bacteria cells could be determined for each mat type using the two oligoprobes.

### 3.5. Image Analysis: Geographical Information Systems (GIS) Analyses

Geographical Information System (GIS) approaches [64,65] were used to analyze CSLM-generated images for spatial patterns of microbial cells and CaCO_3_ precipitates within sections of intact surface mats. Sets of 25–30 images were sampled each from Type-1 and Type-2 mats. Briefly, images were classified using the Feature Analyst extension of ArcView GIS 3.2 [66,67]. Supervised classification was based on selecting representative pixels for each feature (e.g., SRM, cyanobacteria and bacteria). Based on these selections, the program identified all other pixels belonging to the same class. Since the fluorescence signature of cyanobacteria and bacteria was very similar, the two groups could not be separated spectrally. However, since Feature Analyst allows for the identification of linear features even when they are not continuous, all fluorescent filamentous shapes (*i.e.*, cyanobacteria) were identified. Filamentous shapes were subtracted from the image containing both cyanobacteria and other bacteria using a change-detection protocol. Following this classification, areas within images that were occupied by each feature of interest, such as SRM and other bacteria, were computed. Quantification of a given fraction of a feature that was localized within a certain delimited region was then used to examine clustering of SRM close to the mat surface, and later clustering of SRM in proximity to CaCO_3_ precipitates.

For purposes of biological relevance, all images collected using CSLM were 512 × 512 pixels, and pixel values were converted to micrometers (*i.e.*, μm). Thus, following conversion into maps, a 512.00 × 512.00 pixel image represented an area of 682.67 × 682.67 μm. The value of 100 map pixels (approx. 130 μm) that was used to delineate abundance patterns was not arbitrary, but rather the result of analyzing sample images in search of an optimal cutoff value (rounded up to an integer expressed in pixels) for initially visualizing clustering of bacteria at the mat surface. The choice of the values used to describe the microspatial proximity of SRM to CaCO_3_ precipitates (*i.e.*, 0.75, 1.5, and 3 pixels) was largely exploratory. Since the mechanistic relevance of these associations (e.g., diffusion distances) were not known, results were presented for three different distances in a series where each distance was double the value of the previous one. Pearson’s correlation coefficients were then calculated for each putative association (see below).

#### 3.5.1. Ground-Truthing GIS

GIS was used examine spatial relationships between specific image features such as SRM cells. In order to verify the results of GIS analyses, it was necessary to “ground-truth” image features (*i.e.*, bacteria). Therefore, separate “calibration” studies were conducted to “ground-truth” our GIS-based image data at microbial spatial scales.

#### 3.5.2. Calibrations Using Fluorescent Microspheres

An experiment was designed to examine the correlation of “direct counts” of added spherical polymer microspheres (1.0 μm dia.) with those estimated using GIS/Image analysis approaches, which examined the total “fluorescent area” of the microspheres. The fluorescent microspheres used for these calibrations were trans-fluosphere carboxylate-modified microspheres (Molecular Probes, Molecular Probes, Eugene, OR, USA; T-8883; 1.0 μm; excit./emiss. 488/645 nm; refractive index = 1.6), and have been previously used for similar fluorescence-size calibrations [31]. Direct counts of microspheres (and later, bacteria cells) were determined [68]. Replicate serial dilutions of microspheres: *c*, *c*/2, *c*/4, *c*/8, and *c*/16, (where *c* is concentration) were homogeneously mixed in distilled water. For each dilution, five replicate slides were prepared and examined using CSLM. From each slide, five images were randomly selected. Output, in the form of bi-color images, was classified using Erdas Imagine 8.5 (Leica Geosystems AG, Heerbrugg, Switzerland). Classification was based on generating two classes (“microspheres” and background) after a maximum number of 20 iterations per pixel, and a convergence threshold of 0.95 and converted into maps. For the resulting surfaces, areas were computed in ArcView GIS 3.2. In parallel, independent direct counts of microspheres were made for each image. Statistical correlations of direct counts (of microspheres) and fluorescent image area were determined.

#### 3.5.3. Calibrations within Intact Mats

Finally, fluorescent microspheres were added to the surface of Type-1 mats, as an external standard. Experimental additions of microspheres to Type-2 mats could not be accomplished because of the non-sticky nature of the mat surfaces. The mats were then imaged by CSLM and analyzed using the previously-described GIS-based approaches. Following image classification, the areas of microspheres were computed for each image, and correlated with the total number of microspheres counted (via direct counts approach) within the same images. This was designed to examine the ability of the image analysis approach to detect individual bacteria-sized objects (*i.e.*, 1 μm particles) within the complex matrix of natural stromatolite mats.

#### 3.5.4. Microspatial Analyses of SRM and Microprecipitates

SRM activities have been previously implicated in the precipitation of CaCO_3_ within the Type-2 mats of marine stromatolites [10]. Correlative microspatial associations of SRMs and CaCO_3_ precipitates, therefore, were examined over several microspatial scales (approx. 1–5 μm distances) within Type-1 and Type-2 mats. For analyses, paired images were used of the same microspatial regions that were obtained at wavelengths specific to the FISH-probes of SRMs and CaCO_3_ precipitates (488/550 nm = excit/emiss λ).

#### 3.5.5. ^35^SO_4_^2−^-Silver Foils: 2D-Mapping of Sulfate Reducing Activity

Sulfate reducing activity was visualized using ^35^SO_4_^2−^-labeled Ag foil [10]. Ag foil (0.1 mm thickness, 99.99% pure; Sigma-Aldrich, St. Louis, MO, USA) was cleaned using subsequent steps of 30% *w*/*w* hydrogen peroxide and acetone. The foils were allowed to air dry in a class 1000 laminar flow hood. The foils were submersed in a radiolabeled sulfate (Na_2_^35^SO_4_; Perkin-Elmer, Waltham, MA, USA) solution (ca. 0.1 mCi/mL) overnight and allowed to air dry. This treatment was repeated 3–4 times. ^35^SO_4_^2−^-Ag foils were tested for uniform distribution of the label using a BioRad Molecular Imager System GS-525 (Hercules, CA, USA). Freshly collected stromatolite samples were cut vertically and placed on the foil. After 6–9 h of incubation in the dark at 23 ºC, the stromatolite mat samples were removed and the ^35^SO_4_^2−^ washed off the foil using distilled water. The foils (containing ^35^SO_4_^2−^ produced during SR) were kept in the dark and scanned using the BioRad Molecular Imager System GS-525 to visualize a 2-D Ag^35^SO_4_^2−^ distribution. The individual pixels represent an area of ca. 50 × 50 μm, and darker pixels indicate a higher rate of sulfate reduction.

#### 3.5.6. Clustering Analyses of SRMs

The microspatial arrangements of cells relative to each other (*i.e.*, clustering), and changes in relative abundances were examined by examining CSLM images of mat cross-sections. Thirty independent field images from Type-1 and Type-2 mats were examined for each mat type.

#### 3.5.7. GIS

Clustering of SRM cells within the surfaces of Type-1 and Type-2 mats was analyzed using GIS by creating a buffer area extending from the surface of the mat to approximately 133 μm in depth. This surface region was chosen because preliminary examinations showed that most of cells appeared here. Hence our clustering analyses would examine changes in cell distributions within this surface region of the mat. Detection of SRM cells within the buffer area was based on color (as described above) using image classification of FISH-probed cells. A concentric region having a 10 μm dia. was generated around each cell. A cluster of cells represented a group of cells having overlapping concentric regions. Subsequent statistical selection of clusters was subjectively based on cluster areas representing greater than five cells. The size (*i.e.*, area) of each detected cell cluster was measured.

#### 3.5.8. DAIME

Images collected from CSLM were also analyzed for changes in the spatial patterning of SRM cells in both Type-1 and Type-2 mats using the DAIME program [32]. Clustering within images was analysed using the Spatial:Stereology:Spatial arrangement subprogram with Daime. This calculates distances between all objects (*i.e.*, cells) within an image. Analyzed distances (*i.e.*, μm) were expressed as a pair correlation graph. Mean values of pair correlation values >1 indicated clustering at a given distance. Values approximating 1 indicated a random distribution of cells, and values <1 indicated avoidance.

#### 3.5.9. Statistical Analyses

Following spatial analyses, the areas occupied by specific groups of bacteria (e.g., SRM, cyanobacteria) within proximity to the surface, and/or precipitates, cyanobacteria, other bacteria, and cyanobacteria) were tabulated in ArcView GIS (Environmental Systems Research Institute, Redlands, CA, USA). Data were examined using statistical analysis systems (SAS Institute Inc., Cary, NC, USA) software programs, for homogeneity of variances, then a range of statistical tests were used to examine potential differences in microspatial arrangements and associations [69,70]. Appropriate transformations were made, where necessary, to normalize data. Differences in precipitate concentrations between Type-1 and Type-2 mats were examined using a student’s *t*-test. Overall differences in abundances of SRM among Type-1 and Type-2 mats were compared using analysis of variance (ANOVA). Differences in significant treatment effects were distinguished using Bonferroni and Scheffé aposteriori tests. Logistic regression analyses were used to examine clustering changes during transitions from a Type-1 to Type-2 mat. If no significant differences were detectable, mat data was pooled and analyzed as a single category. Pearson’s correlation coefficient analysis was used to determine the specific correlations within given images, of areas occupied by SRM and CaCO_3_ precipitates.

### 3.6. Molecular Phylogenetic Analysis of *dsrA* Genes

For molecular analysis of dissimilatory sulfite reductase *dsrA* genes, 170 mm^3^ cores were removed from the surface of type I and II stromatolites. DNA was extracted from these samples using the Power Biofilm DNA Isolation Kit (MoBio Laboratories, Carlsberg, CA, USA) according to the manufacturer’s protocol and used as template to generate *dsr* gene amplicons. Each PCR reaction consisted of 1.5 mM MgCl_2_, 0.2 mM nucleotides, 0.4 uM of primers DSR1F (5′ACS(C/G)CACTGGAAGCACG-3′) and DSR4R (5′GTGTAGCAGTTACCGCA3′) [38], 1.25 U of Hot start polymerase (Promega), 10 ng of template DNA, and water in a 25 μL volume. PCR conditions were conducted as follows: 95 ºC for 5 min, followed by 35 cycles of 95 ºC for 45 s, 54 ºC for 40 s, 72 ºC for 2 min and a final extension at 72 ºC for 10 min. PCR amplicons were purified with a QIAQuick PCR Purification Kit (Qiagen Sciences, Maryland, MD, USA) according to the manufacturer’s instructions. These purified amplicons were ligated into pCR2.1-TOPO cloning vectors (Invitrogen, Carlsbad, CA, USA), and transformed into One Shot *E. coli* DH5α-T1^R^ competent cells following the manufacturer’s protocol. Transformants were picked and grown overnight at 37 ºC in LB broth containing 50 μg mL^−1^ kanamycin. Plasmids were extracted and purified using QIAprep Spin Miniprep kit (Qiagen Sciences Inc., Alameda, CA, USA), and quantified with a NanoDrop spectrophotometer (NanoDrop Technologies, Inc., Wilmington, DE, USA). Plasmid inserts were sequenced using the M13F (5′GTAAAACGACGGCCAGT3′) and M13R (5′CAGGAAACAGCTATGAC3′) plasmid vector primers at EnGenCore, LLC (Columbia, SC, USA) using BigDye Terminator version 3.1 cycle sequencing kit (Applied Biosystems, Warrington, UK). Resultant sequences were then searched against the GenBank database using BLASTX with default settings. Translated *dsrA* gene sequences from type I and II stromatolites were then aligned with amino acid sequences for the top BLAST hit and other characterized *dsrA* sequences using MUSCLE [71]. Next, a non-rooted phylogenetic tree was constructed using the Maximum Likelihood method based on the Whelan and Goldman model within the MEGA5 [72]. Initial tree(s) for the heuristic search were obtained by applying the Neighbor-Joining method to a matrix of pairwise distances estimated using a JTT model. A discrete Gamma distribution was used to model evolutionary rate differences among sites (5 categories (+G, parameter = 1.2797)). Tree robustness was tested using bootstrap analysis with 1000 replicates.

#### 3.6.1. Extraction and Identification of Quorum Sensing Signals by LC/MS

Culture supernatants of SRM mat isolates were triple extracted in dichloromethane (DCM), dried under N_2_ gas, and reconstituted with 50% acetonitrile, and analyzed by liquid chromatography/mass spectrometry (LC/MS) as previously described [26]. HPLC (150 mm Aquasep C_18_ column, Somerset, NJ, USA) was used to separate AHLs in samples. Detection and identification of AHLs was conducted using a Waters Premier XE triple quadrupole mass spectrometer (Milford, MA, USA) having positive-ion electrospray ionization. The MS was operated in multiple reaction monitoring mode utilizing two characteristic fragment transitions per analyte (*i.e.*, AHL). Natural mat samples, after gentle homogenization, were extracted in a similar manner to culture samples.

## 4. Conclusions

Abundances of SRM and their specific microspatial distributions, derived from image analyses, were used to create possible instruments of discrimination between non-lithifying Type-1 and lithifying Type-2 stromatolite mat communities. In general, Type-1 mats can be characterized as having comparatively lower abundances of SRM cells, and relatively dispersed cell distribution patterns (*i.e.*, limited-clustering of SRM cells). In contrast, Type-2 mats exhibit higher abundances and significant clustering of SRM cells within the uppermost 130 μm of the surface mat. The GIS approach may be most useful for determination of microbial cell patterns and microspatial organization (*i.e.*, areas occupied by cells) over spatial scales of tens to hundreds of microns. Once proper controls were employed, spatial relationships could be rapidly accessed.

Precipitation of micritic crusts are a characteristic feature of both fossil and present-day marine stromatolites. SRM within surface mats may play a defining role in C and S cycling processes that lead to micritic laminae formation in extant marine stromatolites. Our data suggest that development of an abundant and spatially-organized SRM community within the uppermost (oxic region) surface of stromatolite mats was closely aligned with the transition from a non-lithifying (Type-1) to a lithifying (Type-2) state. The progressive development of spatial organization (and high abundances) of SRM in surface mat layers further presents the likely possibility that quorum sensing may be involved in this transition.

## Figures and Tables

**Table 1.** Microspatial proximity between SRMs and CaCO_3_ precipitates in Type-1 and Type-2 mats. Table shows percentages of total bacteria, located within 1.1, 2.2, or 4.4 μm distances from precipitates, which were SRM. Note that wherever precipitates occurred, greater than 82% of bacteria in proximity to precipitates were SRM. (*n* = number of samples analyzed; *p*-value represents results of ANOVA *F*-test). Type-1 mats were found to be significantly different from Type-2 (*p* ≤ 0.05).

% Bacteria near precipitates that were SRMs	Distance of SRM cells from CaCO_3_ Precipitates					

	≤1.10 μm		≤2.20 μm		≤4.40 μm	

	Type-1	Type-2	Type-1	Type-2	Type-1	Type-2
	(*n* = 12)	(*n* = 29)	(*n* = 12)	(*n* = 29)	(*n* = 12)	(*n* = 29)
Mean	82.29 *	95.51	82.71 *	95.78	85.36 *	96.16
(±SE)	±29.92	±7.60	±29.98	±7.37	±25.23	±7.11

* = designates statistical significance at *p* ≤ 0.05.

**Table 2.** Summary table showing acylhomoserine lactones (AHL) extracted from the Type-2 surface mats of marine stromatolites, and from two stromatolite isolates of sulfate-reducing bacteria (SRB). AHLs were identified using mass-spectrometry, and are designated as C4-, C6-, C8-, *etc*., based on the number of carbons in the acyl chain. An *oxo*-C6-AHL indicates a C6-AHL having an *oxo*-group at the C3-position.

Sample	Strain designation	AHLs detected							
**Type-2 mat extract**	-	**C4-**	**C6-**	**C7-**	**C8-**	**C10-**	**C12-**	**C14-**	*oxo-***C6**

*Desulfovibrio vulgaris* (SRB) subsp. *oxamicus*	ATCC 33405D	**C4-**	**-**	**-**	**C8-**	**-**	**-**	**-**	-

**SRB isolates from Type-2 mats**:									
*Desulfovibro* strain 12.1Lac	GeneBank No. DQ822785	**-**	**C6-**	**C7-**	**C8-**	**-**	**-**	**-**	-

*Desulfovibrio* strain H2.3jLac *	GeneBank No. DQ822786	**-**	**C6-**	**C7-**	**C8-**	**C10-**	**C12-**	**-**	*oxo*-**C6**

*Desulfovibrio* strain H2.3jman	-	**-**	**C6-**	**C7-**	**C8-**	**C10-**	**-**	**-**	-

* same strain used in [27].

## Abbreviations

SRM: sulfate-reducing microorganisms
EPS: extracellular polymeric secretions
AHL: acylhomoserine lactones
QS: quorum sensing
CaCO_3_: calcium carbonate
FISH: fluorescence *in-situ* hybridization
GIS: geographical information systems
CSLM: confocal scanning laser microscopy
daime: digital-image analysis in microbial ecology
